# Draft Genome Sequences of 10 Strains of the Genus *Exiguobacterium*

**DOI:** 10.1128/genomeA.01058-14

**Published:** 2014-10-16

**Authors:** Tatiana A. Vishnivetskaya, Archana Chauhan, Alice C. Layton, Susan M. Pfiffner, Marcel Huntemann, Alex Copeland, Amy Chen, Nikos C. Kyrpides, Victor M. Markowitz, Krishna Palaniappan, Natalia Ivanova, Natalia Mikhailova, Galina Ovchinnikova, Evan W. Andersen, Amrita Pati, Dimitrios Stamatis, T. B. K. Reddy, Nicole Shapiro, Henrik P. Nordberg, Michael N. Cantor, X. Susan Hua, Tanja Woyke

**Affiliations:** a Center for Environmental Biotechnology, University of Tennessee, Knoxville, Tennessee, USA; b UT-ORNL Joint Institute for Biological Sciences, Oak Ridge, Tennessee, USA; c DOE Joint Genome Institute, Walnut Creek, California, USA

## Abstract

High-quality draft genome sequences were determined for 10 *Exiguobacterium* strains in order to provide insight into their evolutionary strategies for speciation and environmental adaptation. The selected genomes include psychrotrophic and thermophilic species from a range of habitats, which will allow for a comparison of metabolic pathways and stress response genes.

## GENOME ANNOUNCEMENT

*Exiguobacterium*, belonging to the order *Bacillales* of the phylum *Firmicutes*, was proposed as a new genus in 1983 by Collins et al. (1) and includes 16 species. All these species are Gram-positive, rod-shaped, facultative anaerobes, motile via peritrichous flagella and have been isolated from a wide range of habitats, with temperatures ranging from −12° to 55°C (2). The ability of individual strains isolated under psychrotrophic or thermophilic conditions to grow in the mesophilic temperature range of 15° to 37°C suggests that *Exiguobacterium* species have unique and conserved genetic pathways allowing these organisms to exploit a diversity of temperature-related habitats. In addition, species with close affiliation to modern strains have been isolated from permafrost and ice estimated to be >100,000 years old.

The genome sequences were determined for 10 *Exiguobacterium* strains, including six type strains and four environmental isolates (Table 1). High-molecular-weight genomic DNA was isolated from strains grown overnight at 30°C in tryptic soy broth using the Joint Genome Institute (JGI) modified cetyltrimethylammonium bromide (CTAB) protocol (3). The draft genomes were generated at JGI using Illumina and Pacific Biosciences (PacBio) technologies. Illumina shotgun and long-insert mate-pair libraries were constructed and sequenced using the Illumina HiSeq 2000 platform (4). Filtered Illumina reads were assembled using AllPaths-LG (5). PacBio SMRTbell libraries were constructed and sequenced on the PacBio RS platform, and raw reads were assembled using HGAP version 2.0.1 (6). Genes were identified using Prodigal (7), followed by manual curation using GenePRIMP (8). The predicted coding sequences (CDSs) were translated and used to search the NCBI nonredundant, UniProt, TIGRFam, Pfam, KEGG, COG, and InterPro databases. The tRNAscan-SE tool (9) was used to find tRNA genes, and rRNA genes were identified against models of the rRNA genes built from SILVA (10). Noncoding RNAs were found by searching the genomes for the corresponding Rfam profiles using Infernal (11). Gene prediction analysis and manual functional annotation were performed within the Integrated Microbial Genomes (IMG) platform (12).

**TABLE 1:** Characteristics of 10 *Exiguobacterium* draft genomes

Organism^*a*^	Isolation source	Sequencing and assembly methods	Size (Mb)	G+C content (%)	No. of CDSs	No. of *rrn* operons	No. of tRNAs	No. of Transposases	No. of cold shock genes	GenBank accession no.	No. of contigs
*E. acetylicum* DSM 20416^T^	Creamery waste, UK	PacBio, HGAP	3.28	47	3,323	9	69	40	3	JNIR00000000	3
*E. oxidotolerans* JCM 12280^T^	Fish drain, Japan	PacBio, HGAP	3.09	47	3,053	9	69	34	6	JNIS00000000	3
*E. undae* DSM 14481^T^	Garden pond, Germany	Illumina, AllPaths-LG	3.25	48	3,287	4	56	12	2	JHZV00000000	4
*E. antarcticum* DSM 14480^T^	Microbial mat, Antarctica	PacBio, HGAP	3.22	47	3,250	10	69	89	7	JMKS00000000	7
*E. sibiricum* 7-3	Permafrost, Siberia, Russia	Illumina, AllPaths-LG	3.08	47	3,141	4	48	9	3	JHZS00000000	7
*E. undae* 190-11	Permafrost, Siberia, Russia	Illumina, AllPaths-LG	3.21	48	3,236	5	61	17	3	JHZU00000000	4
*E. aurantiacum* DSM 6208^T^	Potato wash, UK	PacBio, HGAP	3.04	53	3,067	9	67	90	2	JNIQ00000000	2
*E. marinum* DSM 16307^T^	Marine, Yellow Sea, South Korea	Illumina, AllPaths-LG	2.81	47	2,836	8	60	15	2	JHZT00000000	2
*Exiguobacterium* sp. GIC31	Glacier ice, Greenland	PacBio, HGAP	2.97	52	3,005	9	67	38	2	JNIP00000000	2
*Exiguobacterium* sp. NG55	Hot spring, Yellowstone Park, USA	PacBio, HGAP	3.14	48	3,169	11	68	27	2	JPOD00000000	5

a Type strains (^T^) were obtained from the German Collection of Microorganisms and Cell Cultures (DSM) or Japan Collection of Microorganisms (JCM).

The *Exiguobacterium* strains have low G+C contents (average, 48.4%) and vary slightly in their genome size, number of CDSs, and ribosomal RNA (*rrn*) operons (Table 1). Whole-genome sequencing identified 13 transposase families, which is consistent with those found in previous publications (2, 13). The two most abundant transposase families, transposase/inactivated derivatives and IS*605* (*orfB*), are present in all strains. The strains contain two to seven cold shock protein genes (COG1278), one molecular chaperone GrpE (heat shock protein, COG0576), one ribosome-associated heat shock protein (S4 paralog, COG1188), three chaperonin GroEL (HSP60 family, COG0459), three cochaperonin GroES (HSP10, COG0234), and four fatty acid desaturase (COG3239) genes per strain.

The presence of multiple genes encoding stress-responsive proteins may explain the broad temperature range for growth and the ability of the *Exiguobacterium* strains to colonize and thrive in diverse ecological niches.

### Nucleotide sequence accession numbers.

These whole-genome shotgun projects have been deposited in DDBJ/EMBL/GenBank under accession numbers JNIR00000000, JNIS00000000, JHZV00000000, JMKS00000000, JHZS00000000, JHZU00000000, JNIQ00000000, JHZT00000000, JNIP00000000, and JPOD00000000. The versions described in this paper are the first versions, JNIR01000000, JNIS01000000, JHZV01000000, JMKS01000000, JHZS01000000, JHZU01000000, JNIQ01000000, JHZT01000000, JNIP01000000, and JPOD01000000.
